# The role of mucilage envelope in the endozoochory of selected plant taxa

**DOI:** 10.1007/s00114-020-01709-7

**Published:** 2020-12-11

**Authors:** A. Kreitschitz, E. Haase, S. N. Gorb

**Affiliations:** 1 Department of Functional Morphology and Biomechanics, Kiel University, Am Botanischen Garten 9, D-24098 Kiel, Germany; 2 Department of Plant Developmental Biology, Institute of Experimental Biology, University of Wrocław, ul. Kanonia 6/8, 50-328 Wrocław, Poland

**Keywords:** Birds, Endozoochory, Seed mucilage envelope, Pigeons, Seed dispersal

## Abstract

Myxodiaspory (formation of mucilage envelope around seeds and fruits) is a common adaptation to dry habitats known in many families of Angiosperms. The mucilage envelope of some seeds seems to be also a unique morphological adaptation which protects myxospermatic diaspores while passing through the bird’s digestive system. To evaluate the protective potential of mucilage, we fed the diaspores of seven plant species (representing three different mucilage types and three species of non-mucilaginous plants) to pigeons, *Columba livia domestica*. Twenty-four hours later, we collected the droppings of pigeons and examined a total of 18,900 non-destroyed diaspores to check for mucilage presence and germination ability. Out of all the examined diaspores, 4.5% were mucilaginous seeds. Among them, the highest number (12.2–13.5%) of viable diaspores belonged to the hemicellulosic type of mucilage (from *Plantago* species). Only 3.7% of germinating diaspores with pectic mucilage (*Linum usitatissimum*) were collected, and no seeds representing cellulosic mucilage (e.g., *Ocimum basilicum*). For non-mucilaginous plants, we collected only a few individual seeds (0.1% out of 8100 seeds used). We noted that the mucilaginous seeds found in the droppings were able to germinate; however, the germination ability was generally smaller in comparison to the control (i.e., not digested) seeds. Our results revealed that the presence of mucilage envelope has an impact on diaspore dispersal and survivability. With our experiments, we demonstrated for the first time that the mucilage envelope, especially of the non-cellulosic type, supports endozoochory. We also showed that non-mucilaginous seeds can be occasionally dispersed via endozoochory and are able to germinate. The results of our studies can explain the ways of plants distribution at a small, local scale as well as in long-distance dispersal, e.g., between islands or even continents.

**Supplementary Information:**

The online version contains supplementary material available at 10.1007/s00114-020-01709-7.

## Introduction

Plants produce fruits and seeds (diaspores) for reproduction and dispersal. They can be dispersed by animals (zoochory). The transport of diaspores by their attachment to the animal body surface is called epizoochory, whereas dispersal via animal digestive system represents endozoochory. In endozoochoric seed dispersal, many animals play a role as dispersal agents, e.g., bats, birds, lizards, or rats (Beveridge 1964; Clout and Tilley 1992; Herrera 1995; Fukui 1996; Lobova et al. 2003; Vazačová and Münzbergová 2013; Zona 2017; Lovas-Kiss et al. 2018a,b).

Many birds are known to act as dispersers of fruits and seeds. They can eat different types of plant diaspores, e.g., fleshy fruits, berries, fruits with seeds in a sticky matrix, or hard seeds, and disperse them over a range of distances (McEven 1978; Santamaría et al. 2002; Traveset and Riera 2005; Rodríguez-Pérez et al. 2005; Wotton et al. 2008; Bucher and Bocco 2009; Vazačová and Münzbergová 2013; Costa et al. 2014; Orłowski et al. 2016; Dale et al. 2017). Among crop-carrying birds, pigeons are an important vector, and therefore play a significant role in plant long-distance dispersal. The pigeon’s crop is a large organ in which food can be stored for later digestion (Bucher and Bocco 2009). Pigeons can spread both small and big seeds (McEven 1978; Bucher and Bocco 2009). The diaspores eaten by pigeons and other birds are exposed to grinding and acid treatment in the alimentary canal. A firm coating generally protects the diaspores (Yang et al. 2012). Since birds lack teeth, in order to crush their food, they evolved a gizzard, the posterior part of their stomach. The wall of the gizzard comprises a thick layer of smooth muscles, lined internally by a hardened epithelial secretion, the koilin layer. In granivorous birds, the koilin layer together with gravel and other hard particles grinds the food, breaking the diaspore coat—these are called as “grit particles,” and transforming the nutrients into a paste-like condition, which can be further processed and digested (Sturkie 1986; Luttik and de Snoo 2004). If this happened to fruits or seeds, it would be a blind end with respect to plant reproduction and distribution. However, many plants evolved some protection mechanisms preventing their seeds and fruits from being digested. One of such protection mechanisms can be the mucilage envelope surrounding the diaspore (Grubert 1974; Kreitschitz 2009; Western 2012).

Myxodiaspory is the ability to produce the mucilage envelope by plant dispersal units called diaspores (seeds and fruits). This phenomenon is known for numerous groups of plants growing particularly in dry or disturbed habitats (Grubert 1974). The mucilage envelope plays diverse roles beyond reproduction, including creating proper (water) conditions for germination and protecting the diaspore against pathogens or against collecting and from being eaten by small insects (Kreitschitz 2012; Western 2012). Another important function is enabling the dispersal of diaspores in a number of ways. For instance, the adhesive properties of the mucilage can facilitate the attachment of the diaspore to the animals’ bodies, and by this help with their transport via epizoochory. However, adhesion can also act as an anti-dispersal factor anchoring the diaspore to the ground (antitelochory) (Kreitschitz 2012). Additionally, the very low friction of the hydrated mucilage envelope can facilitate the passage of the diaspore through the bird’s gut and should protect the diaspore from digestion (Kreitschitz et al. 2015, 2016), therefore playing a role in endozoochory.

The passage of the diaspore through the animal’s gut can either enhance or inhibit its germination (Willson 1983; Yagihashi et al. 1998; Izhaki and Safriel 1990; Fukui 1996; Traveset et al. 2001). For example, longer retention within the digestive system can cause abrasion to the seed/fruit coat or removal of some parts of the diaspore coating (e.g., pulp), thus enhancing diaspore germination (Beveridge 1964; Barnea et al. 1990; Cochrane et al. 2005). Endozoochory also enables plants to occupy new habitats and maintain genetic diversity (Cochrane et al. 2005; Costa et al. 2014). This can be understood as a process of, e.g., colonization of new habitats by annual, perennial plants and developing adaptations to a new environment. The genetic diversity can also be a result of the formation of new species by, e.g., cross-pollination, polyploidization or mutations, and further speciation processes (Stebbins 1958).

Previously available literature does not provide robust and direct evidence that the mucilage envelope supports endozoochory of plant diaspores. Based on the available literature, it can only be speculated that the diaspores were dispersed due to the presence of mucilage. Many studies focusing on frugivorous and granivorous birds have mainly looked into how endozoochory affects seed germination (Barnea et al. 1990; Rodríguez-Pérez et al. 2005; Vazačová and Münzbergová 2013). Studies describing the morphology of diaspores after having passed the digestive tract are limited to only broad reports dealing mostly on the different degree to which the seed coat is scarified or destroyed after digestion (Beveridge 1964; Barnea et al. 1990; Vazačová and Münzbergová 2013). Furthermore, there are no studies presenting details on the morphology of mucilaginous diaspores after birds’ endozoochory and the function of mucilage in this process.

Mucilage envelope is composed of polysaccharides like pectins, hemicelluloses, and cellulose. In most cases, pectins are the principal constituent of the mucilage’s mass (e.g., in *Linum usitatissimum*), but hemicelluloses can also be dominant in the envelope (e.g., *Plantago* sp*.*) (Naran et al. 2008; Saeedi et al. 2010; Phan et al. 2016). Another variant is the cellulosic mucilage (e.g., in *Ocimum* sp. and *Salvia* sp.), characterized by the presence of cellulose fibrils attached to the seed surface, which interacts with pectins and hemicelluloses forming a net-like structure. This specialized organization of the mucilage supposedly prevents its removal from the seed surface (Kreitschitz and Gorb 2017) in contrast to the mucilage without cellulose skeleton, which can easily be lost (Naran et al. 2008). A protective role of the mucilage is also postulated in endozoochory; however, no detailed analysis has been conducted on the maintenance and function of mucilage envelope in this process. A suggestion that the mucilage envelope could protect diaspores against destruction in the pigeon’s digestive system has been proposed by Vazačová and Münzbergová (2013). The authors collected a few viable seeds of *Plantago arborescens* and *Salvia canariensis* after passing through a pigeon’s digestive system and surmised that the mucilage produced by these seeds likely plays an adaptive role in endozoochory. In other studies, many diverse diaspores have been collected from the bird’s droppings (including pigeons); among them are also some myxospermatic seeds of *Plantago lanceolata* and *Plantago major* (Eber 1962; Cavers et al. 1980). However, no detailed analysis of the mucilage envelope maintenance and function in such diaspores was performed.

In this paper, we aimed to evaluate the impact of passing through the pigeon’s digestive system on the morphology and viability of mucilaginous diaspores, based on our previous studies concerning physical properties of mucilage (Kreitschitz et al. 2015, 2016) and its spatial architecture (Kreitschitz and Gorb 2017). The main aim of our study was to assess the effects of the mucilage envelope on endozoochory. We supposed that diaspores with cellulosic-type mucilage could better withstand the passage through the bird’s digestive system than those lacking the cellulose skeleton. Therefore, the main hypotheses of our study were as follows: (1) the presence of mucilage envelope affects seed survival, expecting higher survival of seeds with a mucilage envelope; (2) the presence of the mucilage envelope affects seed germination after digestion, expecting higher germination after gut passage compared to controls. We also addressed two questions concerning the mucilage envelope morphology and function: (1) whether digestion affects the morphology of the mucilaginous envelope and which of the mucilage type (cellulosic or non-cellulosic) enables better passage through the digestive system? (2) how mechanical abrasion affects the mucilage envelope.

## Material and methods

We selected mucilaginous diaspores of seven plant species based on the following criteria: (1) production of abundant mucilage by diaspores which then can be easily studied, (2) production of mucilage of distinct chemical composition (this is important for the comparative aspect of this study), (3) potentially eaten and spread by birds, (4) potential to be found in nature but also commercially available.

Based on the above criteria, we used commercially supplied mucilaginous seeds of seven plant species: *Linum usitatissimum* L. (flax; Dirk Rossmann GmbH, Germany), *Lepidium sativum* L. (garden cress; Bioforce AG, Germany), *Plantago lanceolata* L. (plantain; Pflanzen-Vielfalt, Germany), *Plantago psyllium* L. (psyllium; Orzeszek sp. z o.o., Poland), *Plantago ovata* Forsk. (blond plantain; Dirk Rossmann GmbH, Germany), *Ocimum basilicum* L. (basil; Fair Trade Handels AG, Germany), *Salvia hispanica* L. (Chia; Dirk Rossmann GmbH, Germany). Additionally, for the comparison, we used non-mucilaginous seeds, which are often admixed to the food for wild and/or domestic birds of the following species: *Amaranthus albus* L. (common tumbleweed; Market Hall, Wroclaw, Poland), *Brassica napus* L. (rape; Faculty of Agricultural and Nutritional Science, University of Kiel), *Nigella sativa* L. (black cumin; Market Hall, Wroclaw, Poland). All of the seeds were bought in the same year (2017) and tested for their viability in germination tests.

### Morphology of mucilage envelope

First, we needed to distinguish between diaspores with cellulosic and non-cellulosic mucilage and to visualize the general morphology and basic components of their mucilage envelope. For this purpose, we stained the diaspores with 0.1% aqueous solution ruthenium red (Sigma-Aldrich) to detect pectins (Braune et al. 1975) and with 0.1% aqueous solution Direct Red 23 (Sigma-Aldrich) to detect polysaccharides containing ß-1,4 linkages, e.g., cellulose (Liesche et al. 2013; Kreitschitz and Gorb 2017). The preparations were documented using a light microscope connected to the camera (Zeiss Axioplan, AxioCam MRc, Carl Zeiss Microscopy, GmbH, Germany) and a Confocal Laser Scanning Microscope (CLSM, LSM 700 Axio Zeiss, Germany) (Direct Red 23 - excitation light 555 nm, emission 560 nm). To detect the presence of the starch grains in the mucilage of *O. basilicum*, we used the solution of potassium iodide with iodine in water. This reaction stains starch grains violet to dark violet (Braune et al. 1975).

Selected properties of mucilaginous seeds (seed dimension, type of mucilage, effect of digestion) used in the experiments and information about the detailed mucilage composition from literature are summarized in Online Resource 1.doc, Table S1.doc.

#### Experiment 1—Diaspore passage through the pigeon’s digestive system

We used a group of racing pigeons (*Columba livia domestica*) from a private colony situated close to the campus of University Kiel. Their regular diet consisted of about 25–30 g mixture of the following seeds (grains): corn 28%, wheat 22%, legumes (mainly peas) 19%, white sorghum 12%, barley 12%, gold millet 3%, red sorghum 2%, black sunflower seeds 2%. Nine adult (˃ 1 year old) pigeons (selected from the whole group), staying in their familiar environment, were used in each experiment. The birds were kept in a room (3.3 m long, 2.0 m wide, 2.5 m high, 2 windows about 1 m^2^ each) of a wooden garden house. Twice daily (in the morning and in the afternoon) they were allowed to fly free for 30 to 60 min and after flying they were called back into their loft. Before the experiment, the pigeons were fed with their regular diet and received water. After 30 min, each pigeon was fed with a briefly moistened 1 ml gelatine capsule (Gelatine-Kapseln C 9 VE, EYDAM, Kiel, Germany) filled with 300 seeds of one tested plant species by putting it deep into the throat. In a case of large seeds, such as those of *L. usitatissimum* and *B. napus*, two or three capsules were filled with the seeds and used at the same time (with few seconds intervals between the individual capsules). Then, the birds were placed individually into their lockable nesting boxes (floor 35 × 49cm, height 39 cm) for 24 h (once) without access to food and water (the nesting boxes were not checked during that time). The bottom of the nesting box was covered with a sheet of paper, and in each box, there was a plastic-coated wire grill on which the pigeon could sit. Droppings fell through the grill onto the paper. After 24 h, the paper sheets with the droppings were transferred immediately to the laboratory for investigating the presence and conditions of seeds, and the pigeons were freed from the nesting boxes. The pictures of droppings with seeds were taken using a stereomicroscope (Leica M205A, Leica DFC420 camera, software LAS V3.8, Leica Microscopy GmbH, Wetzlar, Germany). Then, the seeds obtained in this experiment were utilized for the germination tests (See Experiment 2).

#### Experiment 2—Germination tests with diaspores that had passed through the pigeon’s digestive system and untreated controls

All seeds collected from the droppings (after 24 h) in Experiment 1 were placed in water, separated from each other, and counted. Then, the seeds were immediately moved into Petri dishes covered with wet filter paper, to test the germination energy and strength. Simultaneously, to compare the germination with seeds which were not exposed to digestion, the same number of seeds, as obtained from droppings, was sown for each plant species onto wet filter paper in Petri dishes. The seeds were germinated at room temperature (21–23 °C) in natural day/night cycle characteristic for this part of the year (the experiments were performed from March to September 2017). The germination energy (E - the count of seeds which germinated after a defined short period of time - 5 days) was calculated after 5 days and the germination strength (S - count of seeds which germinated after defined longer time - 21 days, after that time all viable seeds should germinate) after 21 days. We counted seeds, as they germinated, in which the radicle was visible (International Rules of Seeds Evaluation 1997; Kreitschitz 2003; Winiarczyk et al. 2014). Moldy seeds were removed from the experiment to avoid further contamination of the entire sample.

#### Experiment 3—Impact of mechanical scarification on the seed mucilage morphology

To mimic the conditions during seed passage through the digestive system, we put 20 g of small stones, 2–3 mm in size, into a plastic laboratory container (50 ml), added 5 mL of 0.01 M HCl, pH = 1.9–2.2, and 30 seeds of a given taxon. This mixture was put onto a magnetic stirrer (2mag, hotMIX 1, Germany) and mixed for 1 h with 60 rotations per minute at a temperature 39–40 °C. We adjusted the experimental conditions, i.e., stone size, pH, and temperature, close to those of the pigeon’s stomach (Ziswiler and Farner 1972). After this treatment, to examine the mucilage presence and/or distribution, we stained the diaspores with 0.1% aqueous solution ruthenium red and documented the samples using a light microscope connected to the camera (Zeiss Axioplan, AxioCam MRc, Carl Zeiss Microscopy, GmbH, Germany). The non-stained diaspores were also visualized with a stereomicroscope connected to a digital camera (Leica M205A, Leica DFC420).

An additional experiment was performed to examine the mucilage morphology of the diaspores after the passage through the digestive system in detail. This was necessary because the seeds which were used for germination tests (obtained in Experiment 1) could not be stained as the dye could interfere with the germination process. Feeding of pigeons was performed as described previously (in Experiment 1), but in this case, we only used three pigeons for every plant taxon. The diaspores obtained from bird droppings were stained with ruthenium red to detect mucilage and visualize its morphology in the light microscope. Alternatively, some diaspores were air-dried and studied in the scanning electron microscope (SEM). Untreated seeds (i.e., the control group) and air-dried seeds collected from the droppings (from the additional experiment—see above) were glued to the SEM stubs using a carbon-containing double-sided adhesive conductive tape. Samples were coated with gold-palladium (film thickness 10 nm) using a Leica EM SCD 500 High Vacuum Sputter Coater (Leica Microsystems GmbH, Wetzlar, Germany) and visualized in the SEM (Hitachi S-4800, Hitachi High-Tech. Corp., Tokyo, Japan).

### Statistical analysis

To test our central hypothesis, i.e., how many seeds passed the digestive system, and how many from them germinated, we used chi-squared test with R Core Team (2017). The results of the germination energy tests for individual plants and their control samples were analyzed using the Mann-Whitney *U* test (*p* < 0.05) with STATISTICA (13.1, StatSoft, USA).

## Results

### Morphology of mucilage in studied seeds

Hydration of the diaspores revealed the presence of mucilage envelope in all studied seven plant species. A narrow mucilage envelope was characteristic of *L. usitatissimum* and *P. lanceolata*, whereas the five remaining species produced an abundant mucilage envelope. The diaspores of *A. albus*, *N. sativa*, and *B. napus*, which were taken as control species, did not produce a mucilage envelope. Pectins were detected in the mucilage envelope of all studied species, as revealed by staining with ruthenium red (Online Resource 1.doc, Fig. S1.doc). Staining with Direct Red 23 (Online Resource 1.doc, Fig. S2.doc) revealed the presence of delicate, straight cellulose threads in *L. sativum*, whereas thicker fibrils were present in *O. basilicum* and *S. hispanica* mucilage. Weak dispersed signals were detectable in *L. usitatissimum*, revealing the presence of cellulose, but rather as the remnants of the cell wall of mucilage secreting cells than the regular cellulose fibrils of the mucilage envelope. In *P. lanceolata*, the signal of cellulose was very weak, and visible as a thin layer close to the seed surface. In *P. ovata* and in *P. psyllium*, no signal was observed. In addition, we detected the presence of starch grains in the mucilage of *O. basilicum* (Online Resource 1.doc, Fig. S1g.doc).

#### Experiment 1—Diaspore passage through the pigeon’s digestive system

**Fig. 1:**
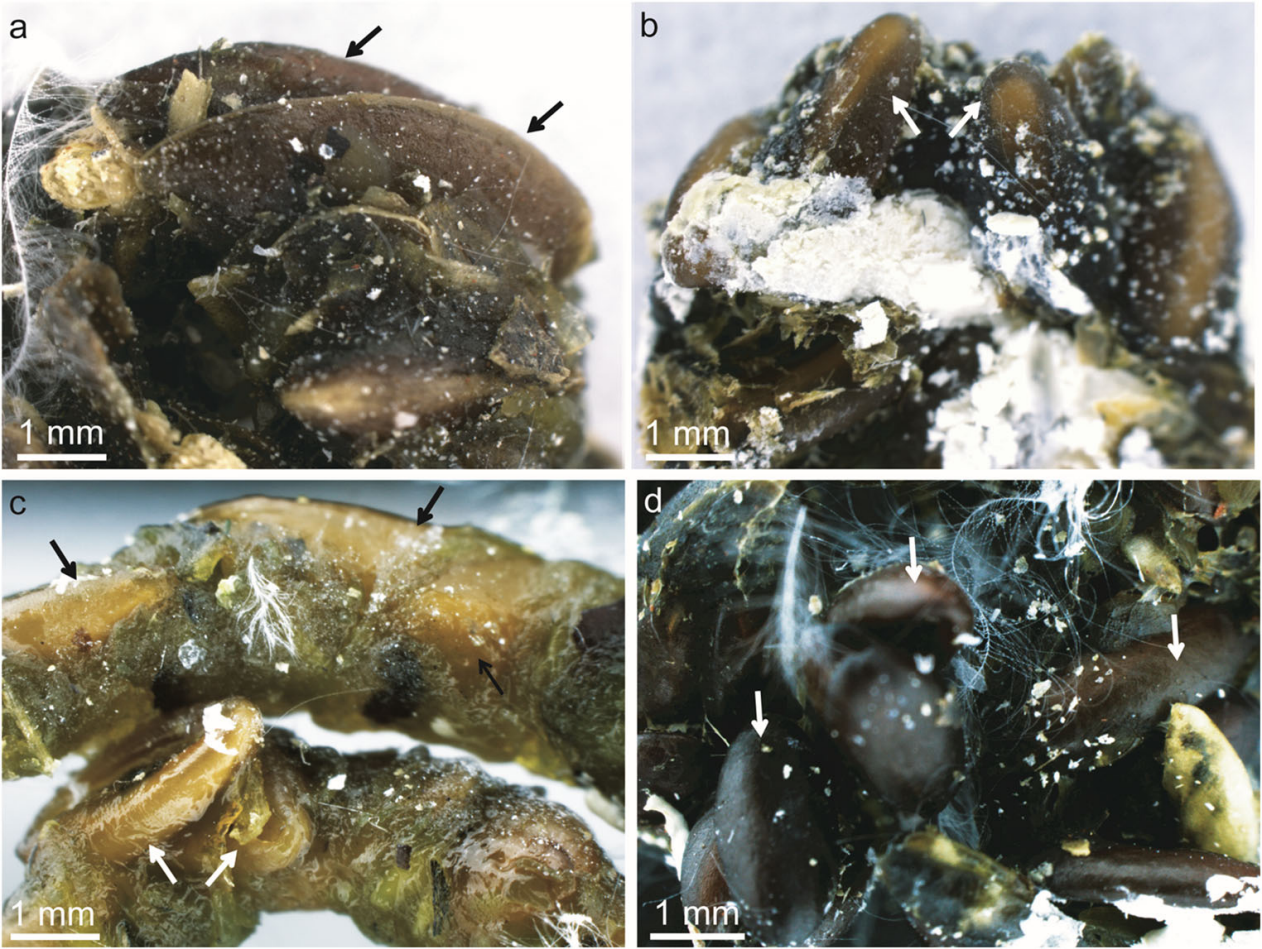
Dropping of *Columba livia domestica* samples with preserved seeds (arrows) glued together by dried mucilage coming from Experiment 1 (diaspores passage through the digestive system) (a) *Linum usitatissimum*. (b) *Plantago lanceolata*. (c) *P. ovata*. (d) *P. psyllium*. In the droppings, different organic materials and the remnants of destructed diaspores were also visible

The mucilaginous diaspores collected from the pigeon’s droppings (Table 1) were glued together by the dried mucilage (Fig. 1) or spread individually. After separating the diaspores from the droppings, it was noticeable that mucilage covering the surface had been maintained to a variable extent. In most seeds, the mucilage envelope was lacking, although there were also diaspores partially or entirely covered by the envelope. The total number of mucilaginous seeds, which passed the digestive system of pigeons (Table 1), was highest for *P. ovata* (13.5%), followed by *P. psyllium* (12.25%), *L. usitatissimum* (3.7%), and *P. lanceolata* (1.7%). Only five seeds of *O. basilicum* passed the digestive system. For two taxa, i.e., *L. sativum* and *S. hispanica*, no seeds were found. Regarding non-mucilaginous seeds, we collected only a few seeds of *A. albus* (0.14%) and *B. napus* (0.11%), and only one seed of *N. sativa* (0.03%) from the droppings*.*

**Table 1:** The count of mucilaginous seeds of seven plant species and non-mucilaginous seeds found in the droppings of *Columba livia domestica*; summary of the germination test. The experimental group refers to seeds which were fed to pigeons; the seeds of the same taxon, which were not fed to pigeons were used as the control group

Plant species (experimental and control samples)		The count of seeds found in the droppings vs. studied control samples	Proportion of seeds found in the droppings (%)^1^	Total count of germinated seeds from those found in the droppings	Energy of germination E (%)^2,^*	Strength of germination S (%)^2,^**	Not germinated seeds-in total (%)^2^
Mucilaginous seeds							
*Linum usitatissimum*	Experimental	100	3.7	4	4	4	96
	Control	100	-	70	70	70	30
*Lepidium sativum*	Experimental	0	0	0	0	0	0
	Control	0	-	0	0	0	0
*Ocimum basilicum*	Experimental	5	0.18	0	0	0	100
	Control	5	-	3	60	0	40
*Salvia hispanica*	Experimental	0	0	0	0	0	0
	Control	0	-	0	0	0	0
*Plantago lanceolata*	Experimental	46	1.7	36	78.26	78.26	21.73
	Control	46	-	46	100	100	0
*Plantago ovata*	Experimental	367	13.59	298	81.19	82.83	17.16
	Control	367	-	358	97.54	98.63	1.08
*Plantago psyllium*	Experimental	331	12.25	188	56.79	67.06	32,93
	Control	331	-	292	88.21	90.33	9.66
Non-mucilaginous seeds							
*Amaranthus albus*	Experimental	4	0.14	3	75	75	25
	Control	4	-	4	100	100	0
*Nigella sativa*	Experimental	1	0.03	1	100	100	0
	Control	1	-	1	100	100	0
*Brassica napus*	Experimental	3	0.11	0	0	0	100
	Control	3	-	3	100	100	0

E* - the germination energy (the count of seeds which germinated after a defined short period of time) was calculated after five days; S** - the germination strength (count of seeds which germinated after defined longer time, after that time all viable seeds should germinate) was calculated after 21 days; E, S - the count of germinated seeds per taxon found in the droppings /the total seeds per taxon found in the droppings (%) (International Rules of Seeds Evaluation 1997; Kreitschitz 2003; Winiarczyk et al. 2014)

^1^Percentages were calculated over the total count of seeds per taxon used in the experiment (2700 seeds per taxon)

^2^Percentages were calculated over the total count of seeds per taxon found in the droppings of *Columba livia domestica*

In summary, 849 mucilaginous seeds (from a total of 18,900 used) passed the digestive system (4.5%) and only 8 non-mucilaginous seeds (from a total of 8100 used, i.e., 0.1%) (*χ*^2^(1) = 354.66, *p* < 0.001) (Online Resource 1.doc, Fig. S3.doc). This confirms our first hypothesis that seeds with mucilage envelope have higher survival than the seeds without mucilage.

#### Experiment 2—Germination tests with diaspores that had passed through the pigeon’s digestive system and untreated controls

Germination tests revealed that the seeds obtained from the droppings were able to germinate (Table 1). The highest germination rate was noted for *P. ovata* with 81%, followed by *P. lanceolata* with 78% and *P. psyllium* with 56%, whereas only 4% of the seeds of *L. usitatissimum* germinated. None of five collected seeds of *O. basilicum* germinated. In tested non-mucilaginous diaspores, only those of *B. napus* did not germinate. Statistically significant differences between the germination energies of seeds, which passed the digestive system, and control seeds were noted only for *L. usitatissimum* (Table 2). The germination energy of non-treated seeds was high for all *Plantago* taxa studied (from 88 to 100%). Non-treated seeds of flax (70%) and basil (60%) germinated considerably better in comparison to other tested seeds. Control non-mucilaginous seeds of all taxa studied reached 100% germination energy.

In summary, we observed lower germination of mucilaginous seeds passing the digestive system, i.e., 61.9% (526/849), in comparison to the control samples, i.e., 90.6% (769/849) (*χ*^2^(1) = 195.56, *pp* < 0.001) (Online Resource 1.doc, Fig. S4.doc). Because the count of the seeds without mucilage which passed the digestive system was very low, we could not test them statistically.

**Table 2:** Results of the Whitney-Mann *U* test used to compare the germination energy of seeds collected from the droppings and control seeds (statistical difference set at *p* < 0.05)

Taxon	Z corrected	*p*	Sample size
*Linum usitatissimum*	− 2.745	0.006*	100
*Ocimum basilicum*	0.723	0.888	5
*Plantago lanceolata*	− 0.693	0.487	46
*Plantago ovata*	− 0.242	0.808	367
*Plantago psyllium*	− 0.854	0.392	331
*Amaranthus albus*	0.080	0.935	4
*Nigella sativa*	0.061	0.951	1
*Brassica napus*	− 1.374	0.169	3

*Statistically significant difference

Seeds of *Plantago* sp. from the droppings were developing a bit slower, i.e., they formed only the root, whereas seedlings that developed from the control seeds had both a root and cotyledons. However, at the end of the experiment, the seedlings were comparable in their morphology. Differences in germination were observed in individual samples (coming from different pigeons). Apart from the four germinating seeds of *L. usitatissimum*, the seeds that had passed the digestive system did not germinate. They also had a very characteristic smell. In one sample of *P. psyllium*, the seeds from the droppings were germinating visibly slower in comparison to the control seeds. Also, all three collected seeds of *A. albus* developed slower as their seedlings developed only the roots, whereas the control seeds developed fully at the same time.

### Morphology of seeds after passing through the pigeons’ digestive system

**Fig. 2:**
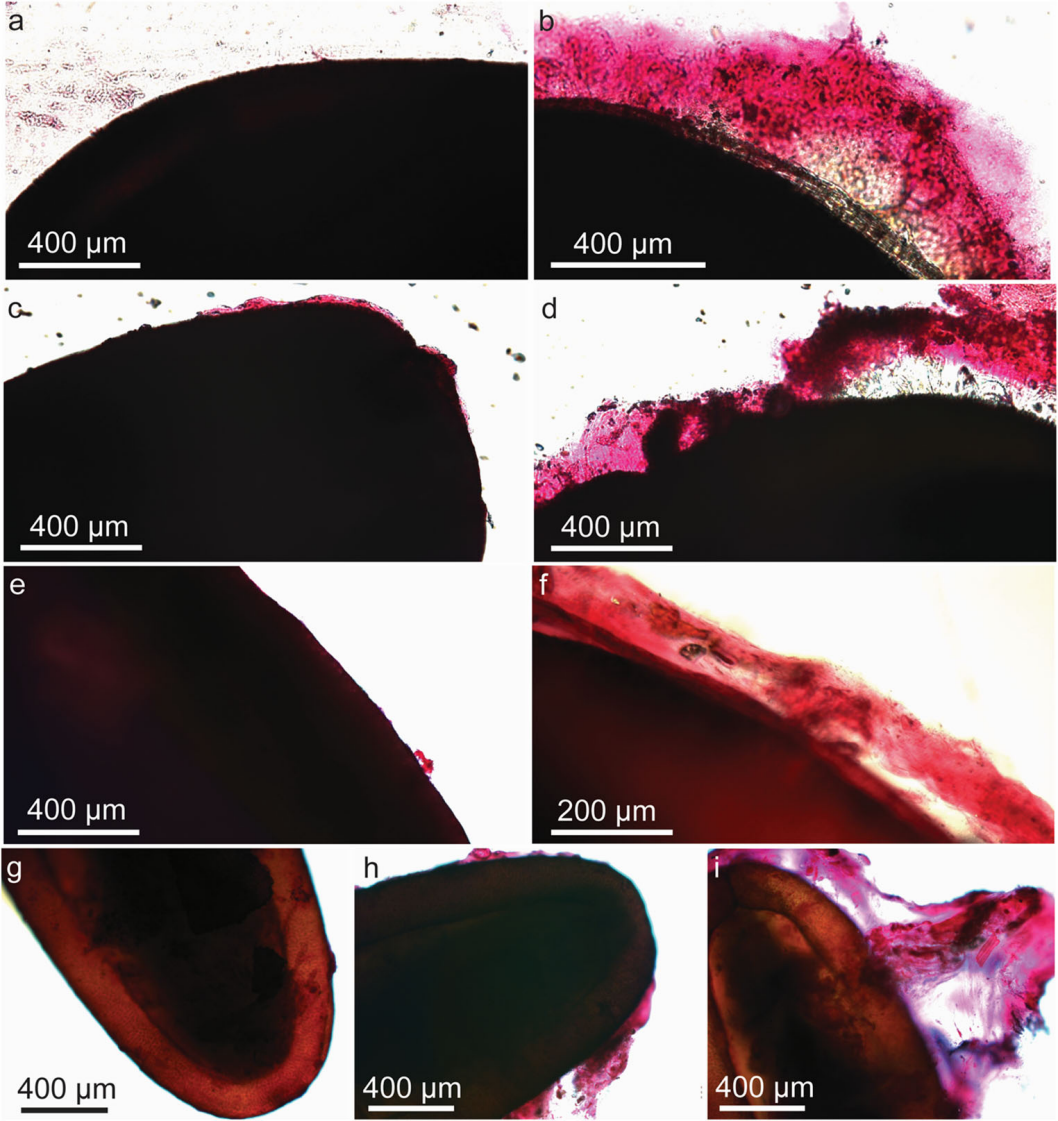
Seeds of four different plant species ((a, b) *Linum usitatissimum*. (c, d) *Plantago lanceolata*. (e, f) *P. ovata*. (g, h, i) *P. psyllium*) collected from the droppings of *Columba livia domestica*, stained with ruthenium red to detect the mucilage and observed in the light microscope. The images show that the diaspores are covered by a mucilage envelope to different degrees. Some do not have a mucilage (*L. usitatissimum*, *P. ovata*), others have only remnants of it (*P. psyllium*), and others still have almost intact envelope present at the surface (*L. usitatissimum, P. ovata*)

For the additional experiments, which were conducted for a detailed examination of mucilage morphology, we found only the seeds of all *Plantago* species, *L. usitatissimum*, and *L. sativum* in the droppings (Fig. 2). For non-mucilaginous plants, only one seed of *B. napus* was collected. The mucilage envelope completely or partially covered the seeds of *L. usitatissimum* and *Plantago* taxa, as revealed by staining with ruthenium red; however, diaspores without mucilage were also present (Fig. 2a, b, e–h). The mucilage envelope morphology was comparable to that described in Experiment 1. Many particles (probably parts of digested food, sand/stone grains) attached to the preserved mucilage envelope were visible (Fig. 4b–d, f). Collected seeds of *L. sativum* revealed a well-preserved mucilage envelope on the entire surface of the seed.

Five seeds of *O. basilicum* obtained from Experiment 1, and which not germinated, were examined in order to describe their mucilage morphology after finishing the germination test. These seeds were covered by well-preserved mucilage envelope, comparable to this of control seeds, with visible cellulose fibrils, starch grains, and many small particles sticking to the envelope.

### Comparison of the surface of untreated (control) seeds and seeds obtained from the droppings by scanning electron microscopy

**Fig. 3:**
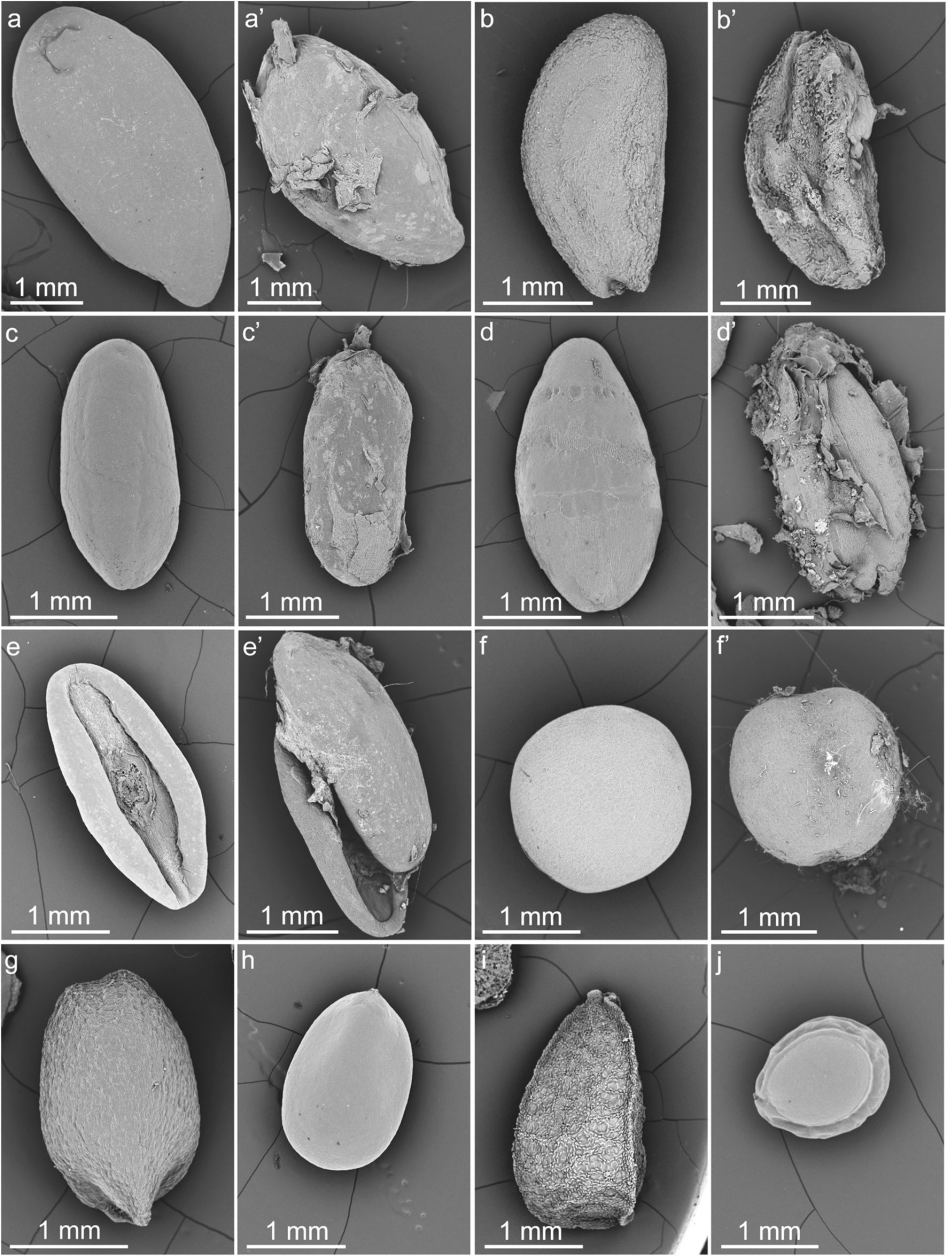
Diaspore surface of control seeds (a–j) and collected from the droppings (a′–f′) of *Columba livia domestica* seeds visualized in scanning electron microscope. (a, a′) *Linum usitatissimum*. (b, b′) *Lepidium sativum*. (c, c′) *Plantago lanceolata*. (d, d′) *Plantago ovata*. (e, e′) *Plantago psyllium*. (f, f′) *Brassica napus*. (g) *Ocimum basilicum*. (h) *Salvia hispanica*. (i) *Nigella sativa*. (j) *Amaranthus albus*. The diaspore surface of passed seeds (a′, b′, c′, d′, e′) was partially destructed and covered with mucilage, to which the remnants of the diaspore coat and other food particles were sticking. In general, the surface of control mucilaginous diaspores was quite smooth with only a delicate sculpture coming from the outlines of mucilaginous cells forming the diaspore coat. The surface of non-mucilaginous seeds was rough (f, i) or smooth (j)

The surface of control mucilaginous diaspores was covered by mucilage secreting cells, which form the outer layer of the diaspore coat. In general, the surface was rather smooth (Fig. 3a–e, g, h), with visible outlines of mucilaginous cells or some wrinkles formed by the cuticle and/or wax, which covered the diaspore. The surface of non-mucilaginous seeds had a delicate surface pattern (*B. napus*, Fig. 3f), with some having a well-visible sculpture (*N. sativa*, Fig. 3i) and others being quite smooth (*A. albus*, Fig. 3j).

The diaspores obtained from the droppings had some mucilage remnants on their surface (Fig. 3a′–e′). The SEM visualization also revealed some breaks and damaged fragments of the diaspore coat. Some small parts of the grit and remnants of non-organic materials were stuck to the surface of the remnants of the mucilage. Damaged diaspore coats exposed underlying layers of parenchymatous or sclerified cells. One collected non-mucilaginous seed of *B. napus* passed the digestive system without visible damages with only some material sticking to the surface (Fig. 3f′).

#### Experiment 3—Impact of mechanical scarification on seed morphology

**Fig. 4:**
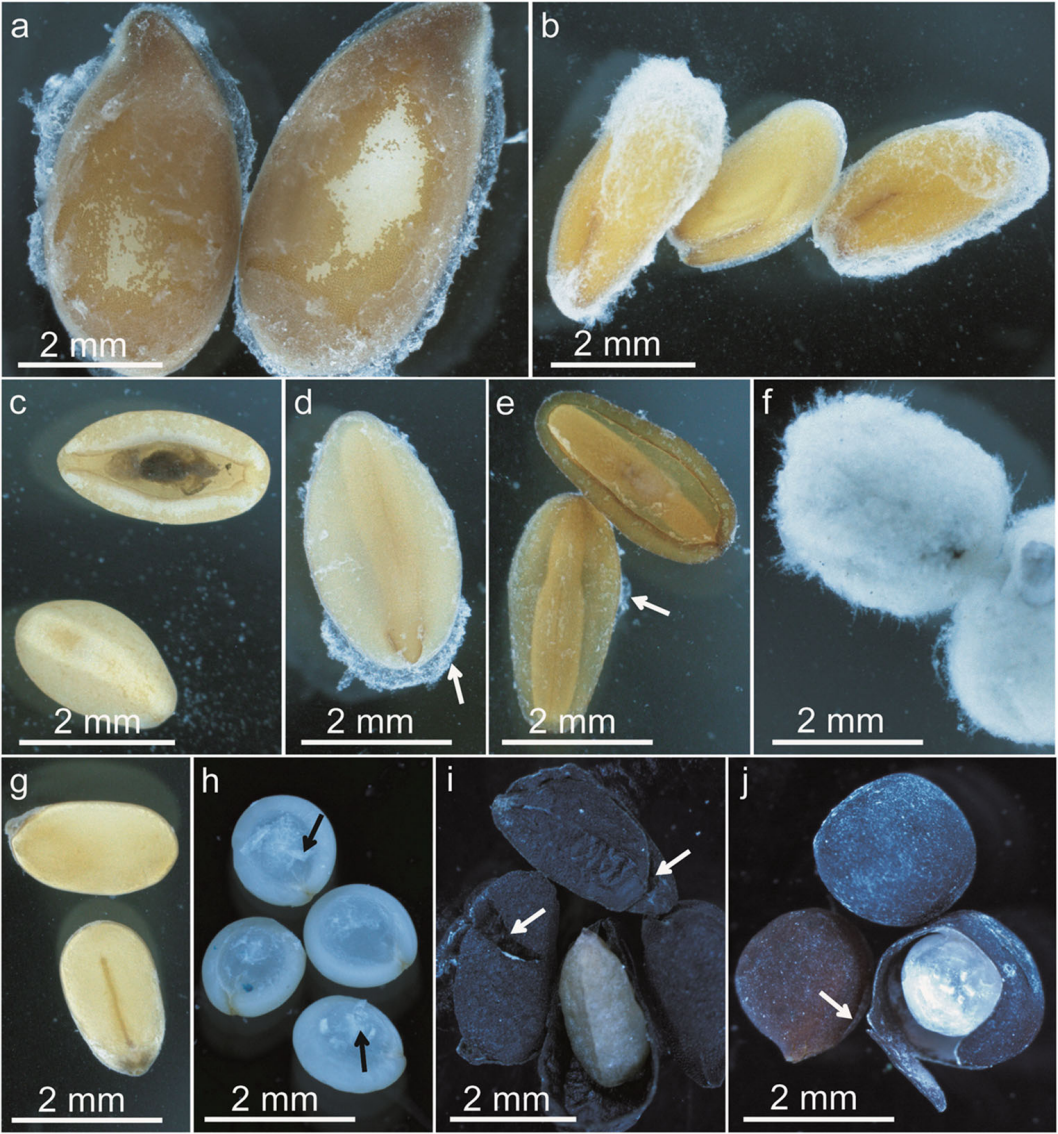
Seeds after mechanical scarification test visualized with a stereomicroscope. After mechanical scarification, the mucilage envelope (a–g, arrow) was lost to varying degrees in diaspores of different plant species. (a) *Linum usitatissimum* and (b) *Lepidium sativum* with some mucilage on the seed surface. (c) *Plantago lanceolata* seeds without mucilage. (d) *Plantago ovata* seeds with some mucilage residues. (e) *Plantago psyllium* seeds with very small residues of mucilage (arrow). (f) *Ocimum basilicum* with the entire envelope preserved on the diaspore surface. (g) *Salva hispanica* the mucilage is completely missing. Non-mucilaginous seeds had different mechanical damages (arrow). (h) Seeds of *Amaranthus albus* had some small scratches on the epidermis. (i) Breaks and fractures of *Nigella sativa* seeds. (j) *Brassica napus* with fractured seed coat and visibly damaged seed coat

In this experiment, we mimicked the passing of a diaspore through the pigeon’s digestive system. After scarification, the mucilage envelopes were preserved to a different degree on the seeds of the examined taxa (Fig. 4a–g, Fig. S5). The seeds of *Plantago* ssp*.* and *L. usitatissimum* almost completely lost the mucilage envelope or were only partially covered with mucilage. Diaspores of *O. basilicum* were completely covered, and those of *Lepidium sativum* were partially covered with mucilage. In these latter taxa, the mucilage layer was easily removable from the seed surface with a pincer. Surprisingly, all *S. hispanica* diaspores were entirely denuded of mucilage. Some small abrasions on the seed surfaces, caused by the stones, were also visible (Fig. 4a), however without any severe damages to the seed coat. In contrast, the non-mucilaginous control seeds displayed some scratches of the epidermis (*A. albus*, Fig. 4 h), as well as some noticeable fractures of the seed coat (*N. sativa* and *B. napus* Fig. 4i, j); however, only five seeds of *B. napus* and 11 seeds of *N. sativa* were totally damaged.

## Discussion

Our results support our initial hypothesis that mucilaginous diaspores can pass the pigeon’s digestive system and still are able to germinate. We showed that mucilage envelope efficiently protects the diaspores against digestion, in contrast to the non-mucilaginous seeds which hardly withstand the passage through the birds’ digestive system. The germination test demonstrated a slightly lower germination ability of mucilaginous seeds which passed the digestive system in comparison to that of (mucilaginous) control samples, which speaks against our initial hypothesis that gut passage of seeds with mucilage can increase their germination ability.

### Survivability of mucilaginous seeds after gut passage

The protective function of the mucilage was further supported by our result that only a small number of non-mucilaginous diaspores survived the passage through the bird’s digestive system (eight out of 8100 tested). We suppose that these eight seeds survived only incidentally, but nevertheless, they maintained the ability to germinate, thus giving the species a chance at dispersal. In contrast, we found that mucilaginous diaspores easily passed through the pigeon’s digestive system and they were found in the droppings in higher numbers, proving that the mucilage envelope can protect seeds against digestion.

The mucilaginous seeds of *Plantago* taxa seem to be particularly well-adapted to endozoochory. They have been found in the dung of numerous mammals, e.g., red deer, cattle and sheep (Cavers et al. 1980; Picard et al. 2016), and birds, including house sparrow, bullfinch, greenfinch, and grey partridge (Cavers et al. 1980; Orłowski et al. 2016; Lovas-Kiss et al. 2018a, b). In addition, diverse seeds from the genera *Brassica*, *Polygonum*, and *Plantago* (mostly *P. major*) have been identified in the droppings of racing pigeons (Eber 1962), and mucilaginous seeds of *Plantago*, as well as *Sinapis* and *Capsella* in the alimentation of the rock dove on the British Isles (Eber 1962; Grubert 1974). The presence of mucilaginous seeds of *Lepidium* sp. (Soons et al. 2016) and *Salvia canariensis* (Vazačová and Münzbergová 2013) and even non-mucilaginous diaspores, also studied in our experiments (*A. albus* and *B. napus*), has also been reported in bird droppings (Soons et al. 2016).

### The germination of mucilaginous seeds after gut passage

We found statistically significant differences in germination between mucilaginous control seeds and mucilaginous seeds, passing the pigeons’ digestive system. Control seeds germinated better than those found in the droppings. This result did not confirm our second hypothesis that mucilaginous seeds after gut passage have a higher germination rate in comparison to control seeds. We argue that the loss of mucilage and some damage to the diaspore coat could sporadically decrease the germination rate, particularly in the case of diaspores which are not adapted to endozoochory. The most noticeable decrease in germination rate was observed in the seeds of *L. usitatissimum*. We postulate that the mucilage produced by *L. usitatissimum* diaspores is an adaptation of the seed for anchoring to the ground rather than a feature related to endozoochoric dispersal. However, it does not rule out the ability of the flax seeds to pass the digestive system as indicated by their presence in the regurgitation pellets of *Corvus frugilegus* (Kitowski et al. 2017).

### The role of mucilage type in endozoochory

The mucilage of *O. basilicum*, *S. hispanica*, and *Lepidium sativum* belongs to the cellulose type characterized by the presence of cellulose fibrils embedded in the mass of pectins. These cellulose fibrils serve as a scaffold for the rest of the mucilage components (pectins and hemicelluloses), and potentially prevent the detachment of the mucilage from the seed surface (Kreitschitz and Gorb 2017). Therefore, we had initially hypothesized that seeds with this type of mucilage should be better protected against mechanical damage or enzymatic digestion in the bird’s digestive system. However, out of the seeds of the three studied taxa with well-developed cellulosic mucilage, only a small number of *O. basilicum* and *L. sativum* and none of *S. hispanica* survived the passage of the digestive system*.* Our results suggest that these species are probably not adapted to endozoochory via birds. Interestingly, *S. hispanica* is a domesticated taxon, and the presence of closed calyxes is a key trait for domestication, as it prevents seed dispersal and effectively eliminates survival of domesticated varieties outside of human cultivation (Cahill 2003). *Salvia* plants drop diaspores directly below the plant (barochory), which is a very common way of dispersal in this genus (Zona 2017). It can be speculated that the presence of cellulosic mucilage in *S. hispanica* is not an adaptation to endozoochory. Similarly, the diaspores of *L. sativum*, with the same type of mucilage, are not dispersed via endozoochory, but more likely via epizoochory as it was observed in New Zealand (Norton et al. 1997). The main dispersal vector can be seabirds to which the mucilaginous diaspores of diverse *Lepidium* taxa can adhere and be transported to various distances (Norton et al. 1997).

Moreover, the cellulosic mucilage envelope can play other roles, e.g., providing the attachment of the diaspore to the soil and preventing their further movement. Cellulose fibrils can be very long and helpful in anchoring diaspores to the substrate (Gutterman and Shem-Tov 1997; Kreitschitz and Vallès 2007). For example, seeds of *Fumana ericifolia* with cellulosic mucilage demonstrated a stronger adherence to the substrate during an experiment with runoff water than seeds of *Helianthemum violaceum* with pectic mucilage (100% vs. 60% seeds remained attached) (Engelbrecht et al. 2014). We suppose that during digestion, cellulose fibrils can stick to small stones present in the gizzard, and thus, they are more easily peeled off from the seed surface after passing through the digestive system.

In addition, the chemical composition and the resulting specific structure of the mucilage can also affect endozoochory. The differences in the count of seeds obtained in this study for three *Plantago* taxa were very noticeable. Mucilage of the *Plantago* taxa is dominated by highly branched hemicelluloses, with natural variation in heteroxylan content and structure (Saeedi et al. 2010; Phan et al. 2016). For example, *P. ovata* mucilage is composed of several layers of hemicelluloses varying in the structure and rheological properties and contains highly substituted forms of heteroxylan (Phan et al. 2016). In contrast, hereroxylans in *P. lanceolata* have fewer substitutions (Phan et al. 2016). The mucilage with highly branched polysaccharides is also specific to the flax seeds. Combination of arabinoxylan (hemicellulose) and highly branched rhamnogalacturonan I (pectin) leads to high viscosity of the flax mucilage (Naran et al. 2008), and could enhance the seed’s ability to attach to a surface. Therefore, we hypothesize that not the presence of cellulose fibrils but rather the differences in the structure and composition of polysaccharides can be responsible for the mucilage adherence to the seed surface during passage through the pigeon’s digestive system.

### Morphology of mucilage envelope after mechanical scarification

To find out if the mucilage envelope can support endozoochory by protecting the seed against chemical and mechanical destruction (i.e., scarification), we performed an experiment mimicking the conditions in the pigeon’s digestive system. Pigeons have two stomachs, which differ in their functions. In the crop, the processes of enzymatic digestion as well as absorption dominate (Sturkie 1986). The muscular stomach (gizzard) is characterized by rhythmic contractions of variable frequency and amplitude and is mainly responsible for the mechanical destruction of food (Ziswiler and Farner 1972). In experiments, mimicking seed passage through the digestive system, grit, and H_2_SO_4_ are commonly used as the main components (Santamaría et al. 2002; Vazačová and Münzbergová 2013; Kleyheeg et al. 2018a; Kleyheeg et al. 2018b). In our experimental setup, we adjusted the conditions of the experiment to mimic those observed in the pigeon’s gizzard, and therefore, we used grit and HCl mixtures, and a magnetic stirrer to simulate the grit movement in the gizzard. Our experiment revealed that the mechanical crumbing caused by stones can result in the total or partial loss of mucilage from the seed surface. However, it seems that the mucilage can well protect diaspores from crushing, as we found diaspores with preserved mucilage envelopes in the droppings. This statement is also supported by the fact that the non-mucilaginous seeds rarely survived passing through the digestive system as they were probably destroyed in the digestive system of the bird, as suggested by the visible fractures and breaks in their seed coat. The difference in the destruction of the seeds could also result from the thickness or hardness of the seed coat; however, some studies have demonstrated that seed coat thickness plays a minor role in determining the intact passage through the duck’s digestive system (Soons et al. 2008). The stomach contractions in connection with the presence of grit can cause severe mechanical damage to the seeds. However, the mucilage envelope, probably due to its low friction (Kreitschitz et al. 2015, 2016), can protect the diaspores against scarification in the stomach. Mucilage provides good slippery conditions for the diaspores, which can more easily pass the digestive system. After passing the bird’s gut, they can be devoid of the mucilage envelope but are still able to germinate as it is clearly demonstrated in our experiments (compare Fig. 2, Table 1).

The flax seeds collected from the droppings and tested for germination smelled very characteristically and were covered by fungi. Flax seeds are known to be rich in oil (El-Beltagi et al. 2007) and we suppose that they were destroyed by digestion, which led to the degradation of lipids or other components stored in the endosperm and this caused the specific smell and further decay by fungi.

### Differences in the seed size

The size of the diaspores has been reported to be a key factor affecting seed survivability during the digestion process. Kleyheeg et al. (2018b) observed that small seeds like those of *Juncus effusus* (0.50 mm long) pass the bird’s gut rapidly (probably also because of the presence of mucilage envelope – AK, (Lazenby 1955)), while large ones like those of *Iris pseudoacorus* (7.66 mm) or *Sparganium erectum* (7.77 mm) are retained for a longer time. The longer retention and large contact area of big seeds can make them more sensitive to digestion and mechanical damage, and consequently can reduce their germination ability (Kleyheeg et al. 2018a, b). In the case of diaspores studied here, we demonstrated that the presence of a mucilage envelope, which is formed immediately after contact with water (also in the digestive tract), plays an important protective role for seeds. Despite the increase of the size of the seed and, consequently the contact area, mucilage envelope prevents the seed from coming into direct contact with digestive enzymes and grit and thus protects the seed from damage. Comparing the seed dimension (see Online Resource 1.doc, Table S1.doc.) from our experiments with the literature data (e.g., Kleyheeg et al. 2018b), we can state that the size of the seed and thickness of the seed coat can play a minor role in the digestion of mucilaginous diaspores. Diaspores selected for our studies were comparable concerning their seed coat characteristics. Besides, the size of the diaspores did not vary significantly among the species studied here. As we saw in the case of non-mucilaginous diaspores, the big seeds of *B. napus* (1.8 × 2.0 mm), *N. sativa* (1.6 × 2.8 mm), and the very small ones of *A. albus* (1.1 × 1.2 mm) passed the digestive system only as individual seeds. It demonstrates that the seed size did not have any influence on seed survival. On the contrary, big diaspores of *L. usitatissimum* (2.1 × 4.4 mm) or *P. ovata* (1.2 × 2.6 mm) survived better due to the protection by the mucilage envelope.

## Conclusions

The results of our study are the first to experimentally support the long-standing hypothesis that mucilage envelope can help in endozoochoric diaspore dispersal. We have shown here that the differences between the mucilage types (cellulosic vs. non-cellulosic) also influence endozoochory. The diaspores with a non-cellulosic mucilage were more tolerant of the passage through the pigeon’s digestive system than those with a cellulosic mucilage. Nevertheless, the cellulosic mucilage appears to play other important roles such as in diaspore attachment to the ground.

## Supplementary Information

ESM 1 (DOCX 8319 kb)

ESM 2 (R 1 kb)

## Data Availability

All data needed to evaluate the conclusions in the paper are present in the paper and/or the supplementary materials in Online Resource 1, 2.
