# Supplementary material for: The role of mucilage envelope in the endozoochory of selected plant taxa
Source: Naturwissenschaften. 2020 Dec 11;108(1):2. doi: 10.1007/s00114-020-01709-7 (PMC7732809; doi:10.1007/s00114-020-01709-7)
Supplement: Supplementary file 1 — (DOCX 8319 kb) [file 114_2020_1709_MOESM1_ESM.docx]

**Supplementary materials**

**The role of mucilage envelope in the endozoochory of myxospermatic plant diaspores**

A. Kreitschitz^1,2*^, E. Haase^1^, S.N. Gorb^1^

*^1^ Department of Functional Morphology and Biomechanics, Kiel University, Am Botanischen Garten 9, D-24098 Kiel, Germany, sgorb@zoologie.uni-kiel.de, euahaase@gmx.de*

*^2^ Department of Plant Developmental Biology, Institute of Experimental Biology, University of Wrocław, ul. Kanonia 6/8, 50-328 Wrocław, Poland, agnieszka.kreitschitz@uwr.edu.pl*

**Fig. S1** Morphology of mucilage envelope of studied diaspores. Staining with ruthenium red revealed the presence of pectins. a. *Linum usitatissimum*. b. *Lepidium sativum*. c. *Plantago lanceolata*. d. *Plantago ovata*. e. *Plantago psyllium*. f-g. *Ocimum basilicum*. g. Coiled cellulose threads with starch grains (arrows) spread between them. h. *Salvia hispanica*


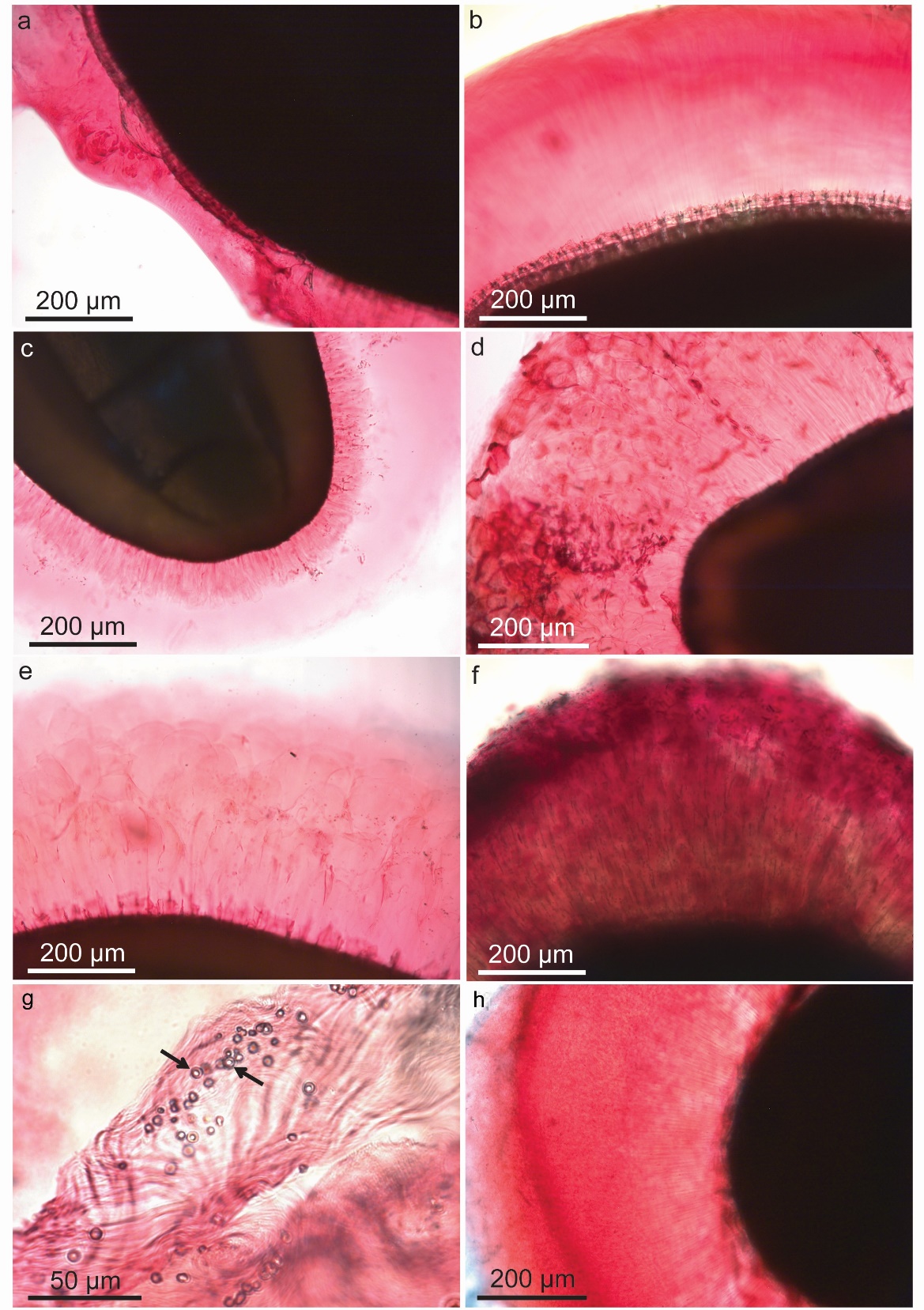


**Fig. S2** Morphology of mucilage envelope of studied diaspores. Labelling of ß-1,4 linkages polysaccharides (e.g. cellulose) using Direct Red 23 (visualized in CLSM). A. *Linum usitatissimum*, signal visible in the remains of the cell walls (arrows) and some mucilage material. B. *Lepidium sativum*, strong signal in the mucilage coming from delicate cellulose fibrils. C. *Plantago lanceolata*, weak signal visible in the mucilage envelope, close to the seed surface. D. *Plantago ovata*, no signal is visible in the mucilage, only some remains of the cell walls are stained. E. *Ocimum basilicum*, coiled cellulose threads in the mucilage. F. *Salvia hispanica*, wavy-shaped, long, thick cellulose threads


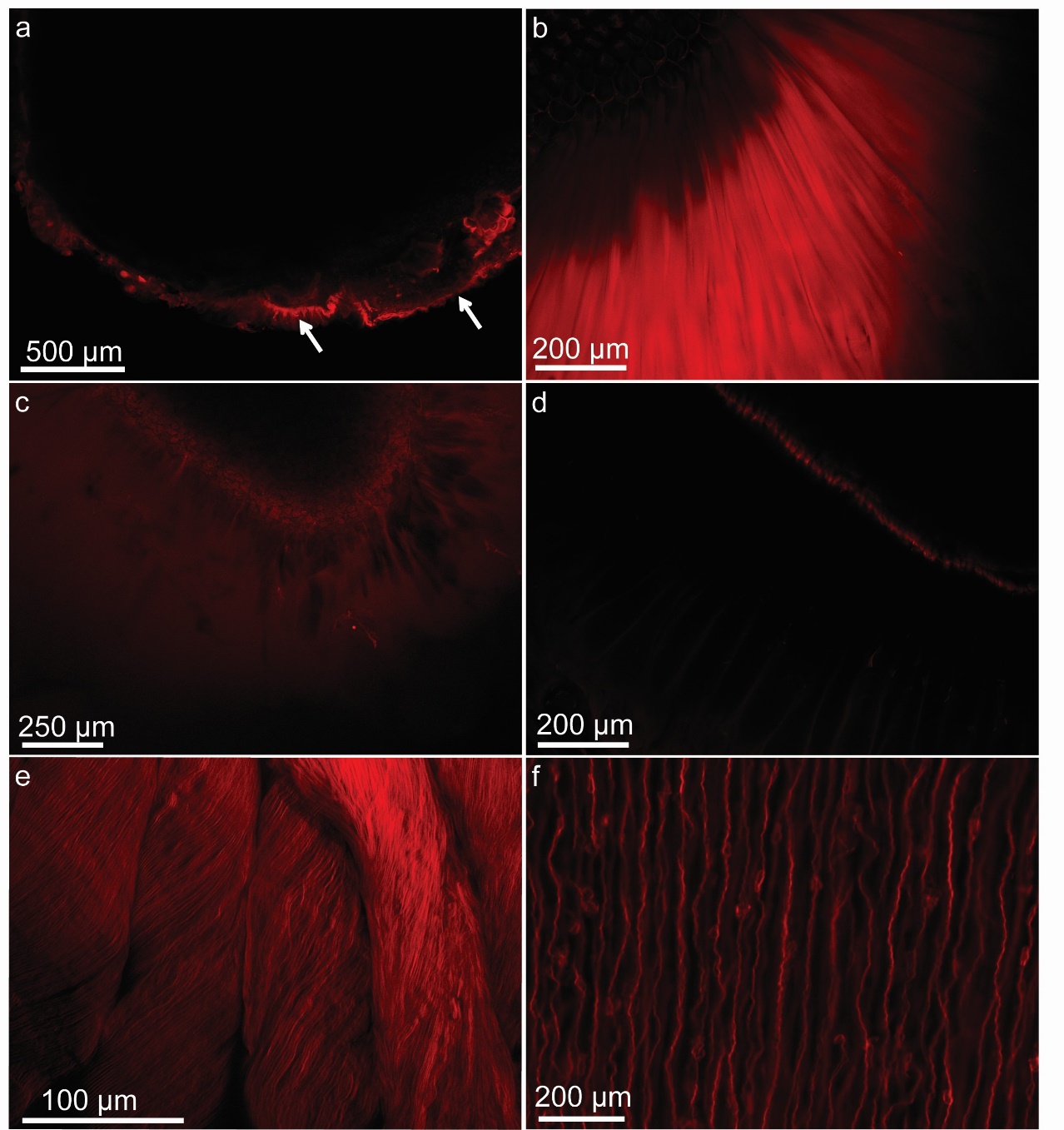


**Fig. S3** Comparison of the total seeds count of all plant species used in the experiment (seven taxa with and three taxa without mucilage) which passed the digestive system of *Columba livia domestica*. From 18 900 used mucilaginous seeds, 4.5% were undigested, whereas only 0.1% of the non-mucilaginous ones were not digested

**
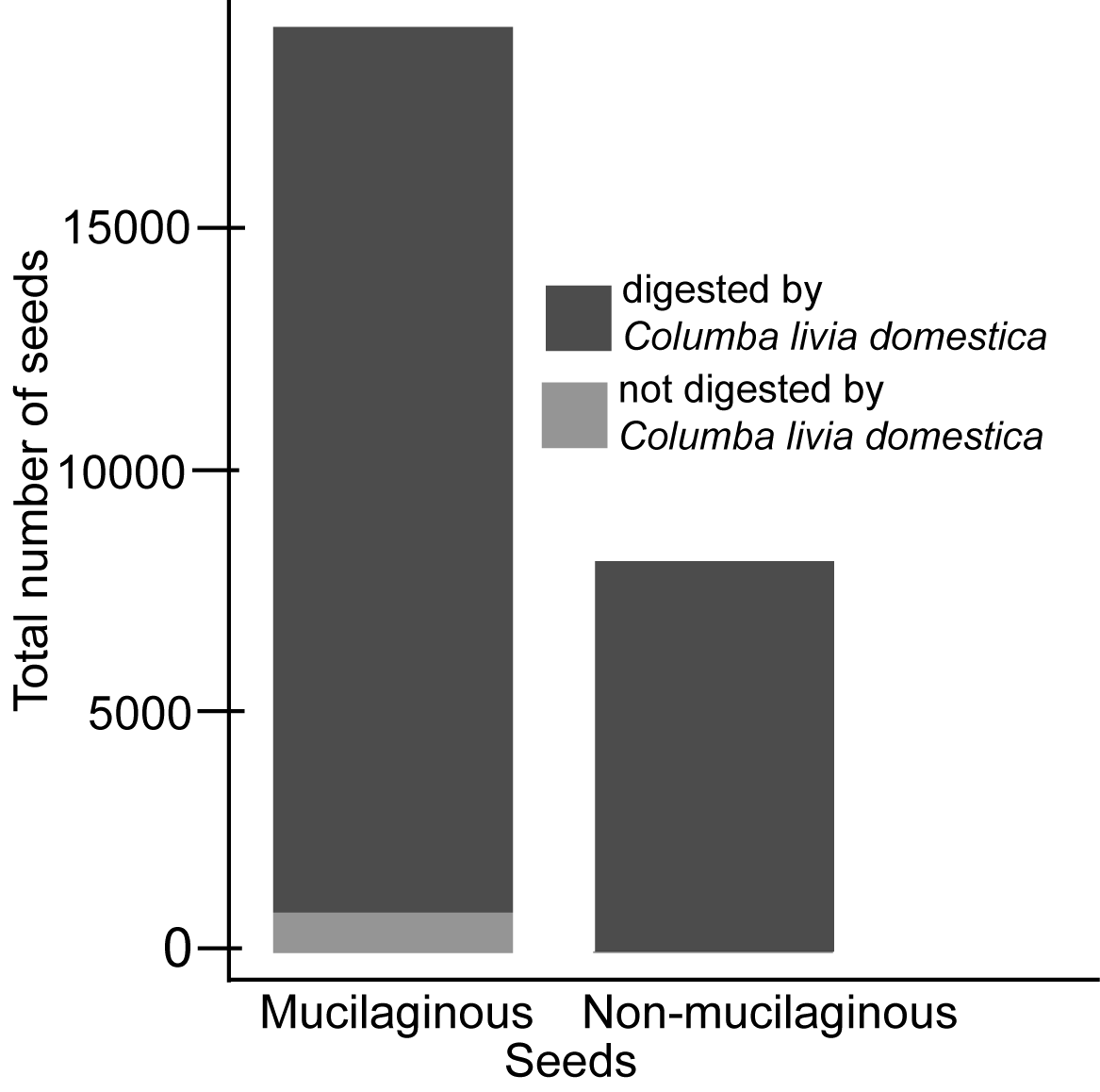
**

**Fig. S4** Comparison of the germination of mucilaginous seeds which passed the digestive system (*L. usitatissimum*, *O. basilicum*, *P. lanceolata*, *P. ovata*, *P. psyllium*) of *Columba livia domestica* and their control samples. From 849 not-digested seeds, 526 (61.9%) germinated. The germination of control sample was higher and reached 769 (90.6%) seeds

**
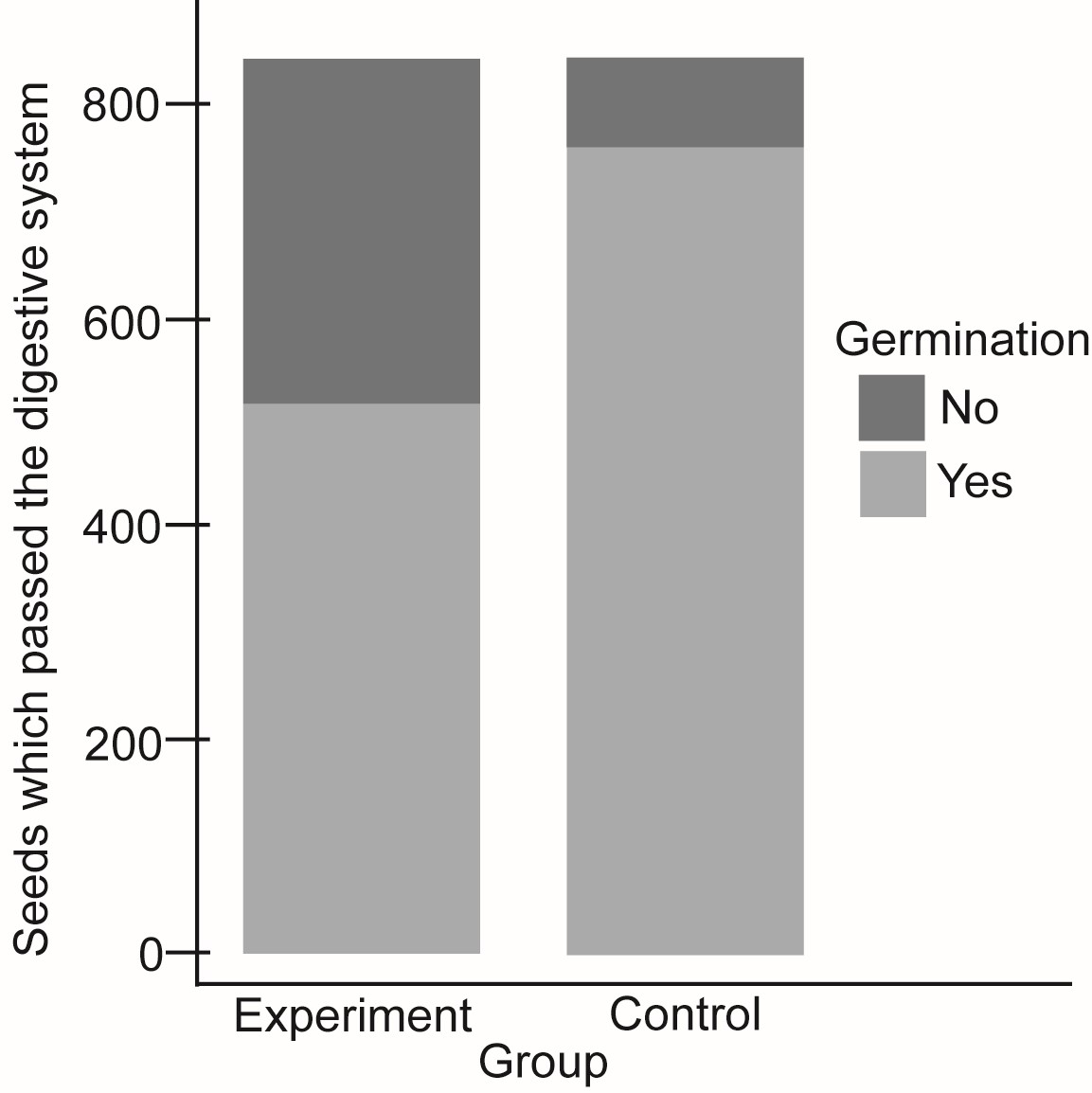
**

**Fig. S5** Mucilaginous seeds after mechanical scarification. A*. Linum usitatissimum*, B. *Lepidium sativum*, C. *Plantago lanceolata*, D. *Plantago ovata*, E. *Plantago psyllium*, F. *Salvia hispanica*, G. *Ocimum basilicum*. The mucilage envelope was lost almost completely from the seeds surface (C, F) or some mucilage remains were still attached to the seed surface (arrows, A, D, E, G). Seeds of *Lepidium sativum* and *Ocimum basilicum* had the best prevented mucilage (B, G). Mucilage envelope was stained with ruthenium red

**
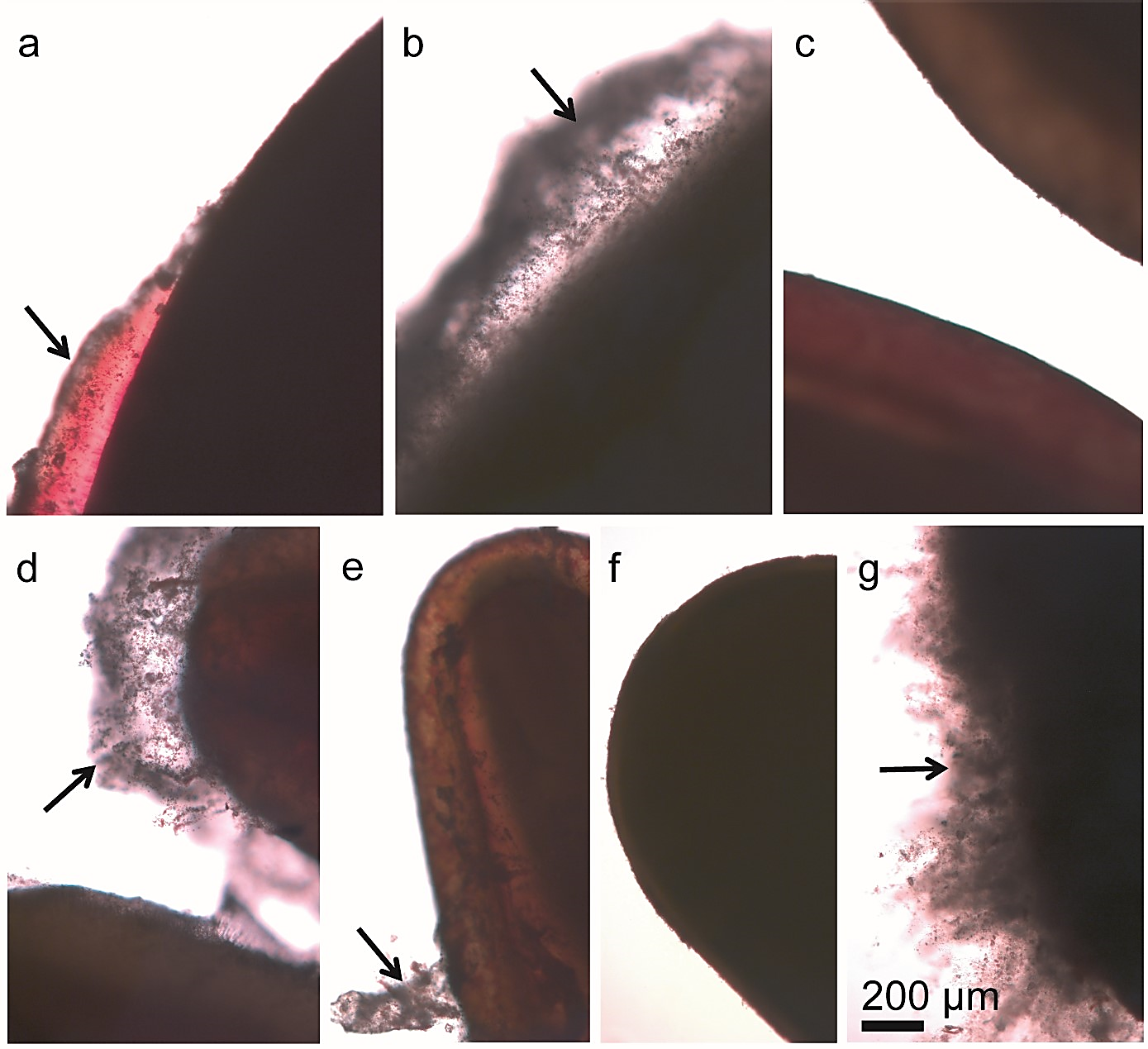
**

**Table S1** The properties of the mucilaginous seeds used in the experiments in reference to literature data

| **Species** | **Seed dimension** | **Type of mucilage** | **Mucilage composition and references** | | **Effect of digestion** | |
| --- | --- | --- | --- | --- | --- | --- |
|  |  |  |  |  | **pigeon** | **artificial** |
| ***Linum sitatissimum*** | 2.1x4.4 mm | Pectic mucilage | The neutral fraction - arabinoxylan (arabinose, xylose, galactose), the acidic fraction (rhamnose, fucose, galactose, galacturonic acid); rhamnogalacturonan I, arabinoxylan; | Muralikrishna ey al. 1987; Naran et al. 2008; | Completely or partially covered with mucilage, without mucilage | Some remnants of mucilage, without mucilage |
| ***Lepidium sativum*** | 1.3x2.6 mm | Cellulosic mucilage | Mannose, arabinose, galacturonic acid, fructose, glucuronic acid, galactose, rhamnose, and glucose, uronic acid, cellulose | Behrouzian et al. 2014; | Completely covered with mucilage | Partially covered with mucilage |
| ***Ocimum basilicum*** | 1.2x2.1 mm | Cellulosic mucilage | Xylan backbone with arabinose, galacturonan, rhamnose and galactose, glucomannan; glucomannan, xylan | Western 2012; Lee et al. 2017 | Completely covered with mucilage | Completely covered with mucilage |
| ***Salvia hispanica*** | 1.2x2.0 mm | Cellulosic mucilage | Xylose, glucose and glucuronic  acid | Muňoz et al. 2012; | No seeds obtained | Without mucilage |
| ***Plantago lanceolata*** | 1.0x2.3 mm | Hemicellulosic mucilage | Less substituted heteroxylan (small amount of cellulose);  arabinoxylomannan-type polysaccharide (mannose, arabinose, xylose, glucuronic acids | Phan et al. 2016; Hesarinejad et al. 2018 | Completely or partially covered with mucilage, without mucilage | Partially covered with mucilage, without mucilage |
| ***Plantago ovata*** | 1.2x2.6 mm | Hemicellulosic mucilage | Highly substituted heteroxylan; arabinoxylans | Fischer et al., 2004, Saghir et al., 2008, Phan et al. 2016; Yu et al. 2017; | Completely or partially covered with mucilage, without mucilage | Partially covered with mucilage, without mucilage |
| ***Plantago psyllium*** | 1.1x2.3 mm | Hemicellulosic mucilage | Arabinoxylans, galacturonic acid; | Western 2012; | Completely or partially covered with mucilage, without mucilage | Partially covered with mucilage, without mucilage |
